# Swedish Version of the System Usability Scale: Translation, Adaption, and Psychometric Evaluation

**DOI:** 10.2196/64210

**Published:** 2025-01-16

**Authors:** Helene Åvik Persson, Charlotte Castor, Nilla Andersson, Mia Hylén

**Affiliations:** 1 Department of Health Sciences, Lund University, Box 117, Sölvegatan 19, Lund, 22100, Sweden, 46 733128650; 2 Department of Care Science, Faculty of Health and Society, Malmo University, Malmö, Sweden; 3 Department of Intensive and Perioperative Care, Skane University Hospital, Malmö, Sweden

**Keywords:** application, Swedish, System Usability Scale, usability, validation

## Abstract

**Background:**

The Swedish health care system is undergoing a transformation. eHealth technologies are increasingly being used. The System Usability Scale is a widely used tool, offering a standardized and reliable measure for assessing the usability of digital health solutions. However, despite the existence of several translations of the System Usability Scale into Swedish, none have undergone psychometric validation. This highlights the urgent need for a validated and standardized Swedish version of the System Usability Scale to ensure accurate and reliable usability evaluations.

**Objective:**

The aim of the study was to translate and psychometrically evaluate a Swedish version of the System Usability Scale.

**Methods:**

The study utilized a 2-phase design. The first phase translated the System Usability Scale into Swedish and the second phase tested the scale’s psychometric properties. A total of 62 participants generated a total of 82 measurements. Descriptive statistics were used to visualize participants’ characteristics. The psychometric evaluation consisted of data quality, scaling assumptions, and acceptability. Construct validity was evaluated by convergent validity, and reliability was evaluated by internal consistency.

**Results:**

The Swedish version of the System Usability Scale demonstrated high conformity with the original version. The scale showed high internal consistency with a Cronbach α of .852 and corrected item-total correlations ranging from 0.454 to 0.731. The construct validity was supported by a significant positive correlation between the System Usability Scale and domain 5 of the eHealth Literacy Questionnaire (*P*=.001).

**Conclusions:**

The Swedish version of the System Usability Scale demonstrated satisfactory psychometric properties. It can be recommended for use in a Swedish context. The positive correlation with domain 5 of the eHealth Literacy Questionnaire further supports the construct validity of the Swedish version of the System Usability Scale, affirming its suitability for evaluating digital health solutions. Additional tests of the Swedish version of the System Usability Scale, for example, in the evaluation of more complex eHealth technology, would further validate the scale.

## Introduction

In the rapidly evolving landscape of global health care, the advent of eHealth technologies has emerged as a transformative force that promises innovative solutions to the multifaceted challenges faced by health care systems worldwide [1,2]. The Swedish health care system is currently transforming along these lines. The use of digital applications and other digital contact methods, collectively described under the term eHealth, is increasing. The World Health Organization (WHO) defines eHealth as “the use of information and communication technologies (ICT) for health.” These technologies include a wide range of systems interventions, applications, and devices such as mobile health and telehealth [3]. There is compelling evidence for the increasing influence of eHealth on the provision of health care globally today and how it is enhancing the efficiency and responsiveness of health systems to meet people’s needs and expectations [3]. It is essential to ensure that health technologies are designed appropriately to meet the needs of end users before deploying them as health interventions [4]. Employing robust evaluation methods to ensure high-level usability has been recognized as a crucial component of good practice for achieving this goal [5].

Usability is defined as “the extent to which a product can be used by specified users to achieve specified goals with effectiveness, efficiency, and satisfaction in a specified context of use” [6]. To determine the potential usability of a digital solution, the System Usability Scale (SUS) has been widely adopted as a standardized evaluative device. In 1996, Brooke [7] published the SUS as an instrument that could easily measure usability. Since then, it has been used to evaluate computer systems, applications, and other digital solutions in a wide range of areas [8-10]. The SUS is a questionnaire, consisting of 10 items each scored on a 5-point Likert scale from “strongly disagree” to “strongly agree.” It is easy to administer and has been shown to generate results with good reliability and validity [7,8,11]. The instrument is free, and no fees are required to use it [7]. With an international reach, it is the most widely used standardized questionnaire for the assessment of perceived usability today [10]. The SUS can be used to evaluate a wide range of usability in products, including digital applications, mobile devices, and web pages [8-10].

However, as psychometric properties are sample dependent, it is essential to evaluate the psychometric properties when using patient-reported outcome measure in new settings or populations [12]. The SUS has been translated into numerous languages such as Chinese [13], Finnish [14], French [15], Hindi [15], Indonesian [16], and Polish [17]. It has undergone psychometric validation [18], including in Arabic [19], Danish [20], Dutch [21], German [22], Italian [23], Malay [24], Persian [25], Portuguese [26], Slovene [27], and Spanish [28]. The psychometric properties that have resulted from these studies show that adapted versions of the SUS are a reliable tool for usability assessments. However, a number of these studies adopted a general focus and examined only the total sum in the test. Only a small number of studies have tested the instrument at an item level [19-28], with none of them in Swedish. This emphasizes the need for a comprehensive testing of a Swedish version of the SUS on an item level. Determining the robustness is critical for ensuring that the measurement instrument has sufficient validity in the proposed context.

Lewis [10] outlines the essential requirements for translating the SUS into multiple languages and conducting validations across diverse countries. In Sweden, one translation of the SUS has been published in a scientific journal [29], although several unpublished versions exist [9]. However, the linguistic discrepancies between these translations give room for ambiguities, and a united translation with a rigorous psychometric testing in a Swedish context is warranted.

In 2016, an initial vision was for Sweden to be “the best in the world” in eHealth by 2025. However, a more realistic view today is that Sweden has made significant progress in this area. Within the European context, Sweden demonstrates a distinct approach to digitalization in health care, emphasizing collaboration and innovation to address specific challenges and opportunities [30].

With this in mind, there is a great need for a nationally united SUS that has been rigorously tested and proven effective. A robust process of evaluating the psychometric properties of a Swedish SUS will foster participatory usability research and ultimately improve the quality of health care services on a broader scale. Although there are different Swedish translations of the SUS, there is still a lack of psychometric testing showing their robustness. Before any Swedish SUS can be recommended for use, both translation and psychometric evaluation of the instrument are necessary. Therefore, the aim of this study was to translate and psychometrically evaluate a Swedish version of the SUS.

## Methods

### Overview

This study consists of 2 steps. In step 1, the SUS was translated and adapted into Swedish. In step 2, the psychometric properties of the Swedish version were tested: data quality, scaling assumptions, acceptability, convergent validity, and internal consistency. The translation, adaptation process, and psychometric evaluation adhered to the COSMIN (Consensus-Based Standards for the Selection of Health Status Measurement Instruments) checklist [31].

### The SUS Instrument

The SUS instrument consists of 10 items (statements). The items are divided equally into positively and negatively worded statements. Requests for a response are graded on a scale of agreement ranging from strongly disagree (1) to strongly agree (5). The score is calculated as follows: for positively worded items (1, 3, 5, 7, and 9), the score is the position on the scale (1-5) minus 1, and for negatively worded items (2, 4, 6, 8, and 10), the score is the position on the scale minus 5. Individual item scores, therefore, can range from 0‐4. The sum of all 10 scores is then multiplied by 2.5, resulting in a total score ranging from 0‐100, with higher numbers representing greater usability [7,8].

To date, factor analysis has not been able to show conclusively whether the SUS consists of one factor (usability) or two (usability and learnability) [11]. Efforts to replicate these findings have led to the conclusion that addressing the instrument as 2 dimensional has no practical or theoretical interest. This study, therefore, treats the SUS as a unidimensional instrument of perceived usability [32].

### Translation and Adaption

According to Brooke’s [7] original formulation of the SUS, no formal permission is needed for translation, and it can be used free of charge. Further, Brooke [7] allows for the possibility to, in any version, exchange the wording of the scale to a word or expression suitable for the situation. The translation process adopted in this study was inspired by Beaton et al [33]. First, the original instrument was translated from English to Swedish by one of the researchers (CC), proceeding from the versions presented by Bangor et al [34] and Lewis [10]. During the process, perceived difficulties and uncertainties were noted. This first version was then reviewed and discussed by the group, consisting of researchers familiar with eHealth, until consensus was reached. Second, an authorized translator, naive to the research field, carried out a back translation on the instrument and the notes taken during the research process. Finally, the research group reviewed all versions of the translation, with all notes attached, and finalized a second version of a Swedish SUS. This version was then compared to other SUS translations within the Scandinavian countries for content validity and the original version for expressions and conceptions that could have been culturally influenced. Following this last step, the group then decided upon a final version by consensus. The response options were structured in the same way as the original questionnaire, although the phrases “strongly disagree” and “strongly agree” were exchanged for the Swedish equivalents of “totally disagree” and “totally agree.”

### Psychometric Evaluation

#### Sample

The evaluation of the psychometric properties was carried out in conjunction with a larger intervention study evaluating eHealth, the eChildHealth tablet study [35]. Parents of children with a range of illnesses and health conditions who were patients in the pediatric department of a level-3 hospital in the south of Sweden were invited to take part. A total of 66 parents to 52 unique children gave informed consent and were included in the eChildHealth tablet study. Of these parents, 62 provided information on the SUS, resulting in a total of 82 measurements.

#### Data Collection

Parents were introduced to an app on a tablet computer through which they could communicate with health care staff after their child had been discharged from hospital. The app made it possible to continue to communicate with hospital staff whom the parents knew well, through chat messages, sending photos, video calls, and predesigned questionnaires. Using a questionnaire, data on various aspects of eHealth were collected for each parent. The SUS was one aspect, and the eHealth Literacy Questionnaire (eHLQ) [36] was another. A study-specific questionnaire was used to collect demographic data such as age and level of education. Between October 2022 and October 2023, the 66 parents were included in study. Data were collected after 1‐2 weeks of use and at a second time point for those participants who used a tablet for more than 1 month. These parents constitute the eligible participants for this study. A sufficient adequate sample size of approximately 80 measurements was based on recommendations from COSMIN [31] and Beaton et al [33].

#### Data Analysis

The psychometric properties of the SUS were analyzed with IBM SPSS Statistics for Windows (version 28.0, IBM Corp). Descriptive statistics (mean, SD, and percentage) were used to visualize participants’ characteristics along with data quality, scaling assumptions, and acceptability. Construct validity was evaluated by convergent validity, and reliability was evaluated by internal consistency.

For data quality, use within the clinical setting was determined by item nonresponse and missing scale scores, as they reflect the acceptance and understanding of a measure [37]. Data quality was determined as high if the percentage of missing data per item was low (<10% acceptable). In this study, there were missing data in 5 items (ranging from 1/81, 1% to 3/81, 4%), representing high data quality. Participants with more than 3 unanswered questions were excluded (n=1), while mean imputation was carried out for those with 1 or 2 missing items (n=6, all missing 1 item) [38]. This resulted in a total of 81 measurements being included in the final analysis.

Regarding scaling assumptions, the dimensionality of the SUS has been evaluated previously [32]. In accordance with these studies, this study assumes that the SUS is to be treated as unidimensional (all items measure the same construct). Instruments composed of Likert-scale items can be summarized if they have similar means and SDs. Furthermore, item-total correlations (the correlation between each individual item score and the total score) would indicate if all items contribute equally to the total score. In line with Hobart et al [37], item-total correlations with values of *r*≥0.3 were regarded as sufficient for summing up the items to a total sum score.

To evaluate the acceptability of score distributions, ceiling and floor effects along with skewness were calculated. Ceiling and floor effects were regarded as present if they exceeded 90%, that is, the percentage of responses for the lowest and highest scores. Skewness statistics should preferably be within the range of −1 to +1 [37].

Construct validity was explored through convergent validity. It was evaluated with the correlation between the high total sum of the SUS and one of the domains of the eHLQ [36], with the hypothesis that it would correlate with the total score of the SUS. Both instruments were distributed to the parents at the same time points. The eHLQ has already been translated, adapted, and validated within a Swedish context [39]. The eHLQ consists of 7 domains across 35 items, with each domain being extractable and treatable as a separate scale. Responses to each domain on the eHLQ are recorded on a Likert scale from 1 (strongly disagree) to 4 (strongly agree). The eHLQ was designed to be used to understand and evaluate people’s interaction with digital health services [36,39]. The research group independently reviewed the eHLQ and, after discussion, decided to use domain 5, measuring the motivation to engage with digital services. The correlation between the instruments was assessed using the nonparametric correlation coefficient of Kendall Tau-b. With regard to the comparative instrument not being used in total (only 1 domain), the correlation was regarded as acceptable if it was moderate or greater (>0.3) [40] and with the level of significance being *P*<.05.

Internal consistency—how items are related to each other—was explored according to the indicators recommended by Hobart et al [37]: corrected item-total correlations and Cronbach α. The cutoffs were >.04 for acceptable corrected item-total correlations and >.8 for Cronbach α [37,41]. The internal consistency was completed with an SEM to analyze the measurement error of the instrument. The SEM represents the smallest difference in scores and indicates a change on a group level. SEM was analyzed with SD_baseline_ × √1 – reliability and complemented with a CI 95% [12].

### Ethical Considerations

This study was conducted in accordance with the Declaration of Helsinki [42] and approved by the Swedish Ethical Review Authority (2021‐05077). The invited parents were recruited through gatekeepers at the pediatric department. Information regarding the study was initially provided orally and then followed up by written information, before written informed consent was obtained by a study nurse. Data were handled confidentially, and participating parents were able to quit without any explanation or impact on the care their children received. Participation in the study was voluntary, and no financial or other form of compensation was provided to the participants.

## Results

### Translation and Adaption

Both translations (forward and backward) were similar and did not differ substantially. Overall, the conformity of phrasing was high, with some discrepancy for phrasing in statements 2 (I found the system unnecessarily complex) and 8 (I found the system very cumbersome to use). In statement 2, the word “complicated” was suggested in the back translation from the Swedish word “komplex.” In statement 8, the word “awkward” was suggested instead of the original word “cumbersome.”

All such discrepancies were discussed within the research group and a consensus was reached for the final version. The final step, comparing the Swedish version to other Scandinavian versions, generated no further changes. Table 1 shows the original English version and the final Swedish version of the SUS.

**Table 1.** Original English version [7] and the proposed Swedish version of the System Usability Scale, including (1) response alternatives and (2) statements.

Component	English version	Swedish version
Response alternatives	1 - Strongly disagree 2 3 4 5 - Strongly agree	1 - Instämmer inte alls 2 3 4 5 - Instämmer helt
Statements	I think that I would like to use this system frequently I found the system unnecessarily complex I thought the system was easy to use I think that I would need the support of a technical person to be able to use this system I found the various functions in this system were well integrated I thought that there was too much inconsistency in this system I would imagine that most people would learn to use this system very quickly I found the system very cumbersome to use I felt very confident using the system I needed to learn a lot of things before I could get going with this system	Jag tror att jag skulle vilja använda denna applikation ofta Jag uppfattar denna applikation som onödigt komplex Jag tycker att denna applikation är enkel att använda Jag tror att jag skulle behöva stöd för att kunna använda denna applikation Jag upplever att de olika funktionerna i denna applikation var väl integrerade Jag tycker att applikationen är inkonsekvent Jag föreställer mig att de flesta personer skulle lära sig att använda denna applikation väldigt snabbt Jag upplever denna applikation som krånglig Jag känner mig trygg med att använda denna applikation Jag behövde lära mig många saker innan jag kunde komma igång med denna applikation

a In this study, the Swedish instruction was as follows: "Markera det alternativ som bäst beskriver din reaktion för applikation i surfplatta idag," meaning “Please indicate your agreement with the following statements, one at a time.”

### Psychometric Evaluation of the Swedish Translation

A total of 62 individuals were included in this study. Of these, 20 answered the questionnaire, including the SUS, twice. This resulted in a total of 81 measurements included in the analysis. Demographic data for the participants are shown in Table 2.

**Table 2.** Demographic data of participants included in the study (N=62).

Demographic data	Values	
**Age (years), median (range)**	33 (22‐52)	
**Gender, n (%)**		
Female	41 (66)	
Male	20 (32)	
Unknown	1 (2)	
**Marital status, n (%)**		
Married	33 (53)	
Living together	28 (45)	
Divorced or separated	1 (2)	
**Education level, n (%)**		
High school	18 (29)	
College or university	42 (68)	
Other	2 (3)	
**Born in Sweden, n (%)**		
Yes	52 (84)	
No	6 (10)	
Unknown	4 (6)	
**First language**		
Swedish	56 (90)	
Other	6 (10)	

#### Data Quality

The percentage of missing data per item was low (ranging from 0/81, 0% to 3/81, 4%) across all items. A tendency could be seen for a higher percentage within the 3 highest steps of the scale, resulting in 5 items having 0% in the lowest points of the 5-point scale (Table 3).

**Table 3.** Missing data (n and %) and item frequency distribution (%) of answers per response alternative in each question of the Swedish System Usability Scale (n=81). The item “0” equals the response of “1” on the scale, etc.

Item	Missing data, n (%)	Item frequency distribution, n (%)				
		0	1	2	3	4
1	0 (0)	1 (1)	5 (6)	21 (26)	36 (44)	19 (23)
2	1 (1)	0 (0)	4 (5)	16 (20)	9 (11)	52 (63)
3	0 (0)	1 (1)	0 (0)	10 (12)	24 (29)	47 (58)
4	2 (2)	0 (0)	0 (0)	6 (7)	16 (20)	58 (71)
5	0 (0)	2 (2)	2 (2)	21(26)	32 (39)	25 (31)
6	3 (4)	2 (2)	3 (4)	21 (25)	13 (16)	40 (49)
7	1 (1)	0 (0)	1 (1)	4 (5)	34 (42)	42 (51)
8	2 (2)	1 (1)	1(1)	5 (6)	10 (13)	63 (77)
9	0 (0)	0 (0)	0 (0)	6 (7)	28 (34)	48 (59)
10	0 (0)	0 (0)	1 (1)	4 (5)	14 (17)	63 (77)

#### Scaling Assumptions

Item means ranged from 2.82 to 3.70 (Table 4). The item-total correlations showed that each item contributed substantially to the total score with correlations ranging from 0.454 to 0.731 (Table 5), thus indicating that the scale can be summarized. The total sum for the SUS in the data ranged between 50 and 100 (mean 84, SD 13).

**Table 4.** Item descriptive statistics for the Swedish version of the System Usability Scale (n=81).

Item	Score, mean (SD)		Skewness
1	2.82 (0.904)		−.550
2	3.35 (0.964)		−1.094
3	3.41 (0.800)		−1.490
4	3.65 (0.618)		−1.585
5	2.93 (0.940)		−.766
6	3.09 (1.076)		−.876
7	3.44 (0.652)		−1.038
8	3.66 (0.762)		−2.682
9	3.51 (0.633)		−.943
10	3.70 (0.622)		−2.203

**Table 5.** Item-total statistics for the Swedish version of the System Usability Scale (n=81).

Item	Scale mean if item deleted	Corrected item-total correlations	Cronbach α if item deleted
1	30.80	0.463	.847
2	30.30	0.731	.820
3	30.22	0.534	.840
4	30.00	0.657	.833
5	30.72	0.479	.847
6	30.57	0.463	.852
7	30.21	0.454	.846
8	29.99	0.687	.827
9	30.12	0.675	.831
10	29.96	0.585	.837

#### Acceptability

As indicated by the item mean score (range 2.82-3.70; Table 4) and item-frequency distribution (Table 3), the instrument showed acceptability. For 3 items (4, 8, and 10), the item frequency was above 70%, which is still within the acceptable range for the absence of a ceiling effect. Skewness statistics were below or near the acceptable range of −1 for a total of 7 items, thus indicating that the distribution was excessively skewed (Table 4).

#### Convergent Validity

Convergent validity was evaluated with the correlation between the total sum of the SUS and the total sum of domain 5 in the eHLQ. As expected, there was a positive correlation between the total score of the instruments (correlation coefficient 0.305), which was significant (*P*=.001) and supported the construct validity of the Swedish version of the SUS.

#### Internal Consistency and Measurement Error

Cronbach α for the scale was .852. Corrected item-total correlations were between 0.454 and 0.731, as shown in Table 5, indicating internal consistency for the different items. For all items except item 6 (I thought there was too much inconsistency in this system), the α value if the item deleted was lower than the Cronbach α for the scale. The SEM was 5.05 (95% CI –4.84 to 14.954) points for the Swedish version of the SUS.

## Discussion

### Principal Findings

This study presents a new Swedish version of the SUS that is psychometrically tested. This study seeks to establish the new Swedish version of the SUS as a reliable and valid instrument for assessing system usability. Overall, the psychometric testing showed high data quality, good scaling assumptions, high internal consistency, and fair convergent validity. Together, these analyses support the validity and reliability of the new Swedish version of the SUS.

The translation process of the new Swedish version of the SUS was executed incrementally, both within and outside the research team, involving an authorized translator who conducted a back translation. This method facilitated thorough scrutiny and comparison of the translation from multiple perspectives. The approach also helped reduce the risk of bias and improved the scale’s validity by incorporating multiple viewpoints [43].

The psychometric testing regarding internal consistency showed that the Cronbach α values were satisfactory and that all items contributed to the instrument’s total score. This indicates that the Swedish translation of the SUS is a stable instrument to use in a Swedish context.

Convergent validity showed fair correlation between the SUS and domain 5 of the eHLQ, which measured the motivation to engage with digital services (I find that digital technology support me in taking care of my health). This supported our hypothesis that a generally positive attitude toward digital solutions would correlate with a high usability score. This hypothesis is also in line with previous research, which shows that positive expectations directed toward a product generate positive subjective usability ratings [14].

There was an indication of a ceiling effect for 3 items on the Swedish version of the SUS (items 4, 8, and 10). They presented above 70% in item frequency distribution and a high mean total score, which is in line with studies indicating a generally high score for the SUS [10]. Previous studies have also shown ceiling effects of the SUS but only for the total score [15,44], thus the responses on the different items can not be justly compared between studies. This study, however, explores the SUS in much more detail and in line with recommended psychometric evaluations, as it explores the psychometric properties for each item separately [12,37]. Further, the distribution was excessively skewed. This could be an expected result of the nonnormal distribution, since negatively skewed variables are assumed to have a ceiling effect [37,45]. Also, the robustness of skewed distribution as the sole indicator of ceiling effect has been questioned [45].

However, based on the skewness result of the items, the question arises as to whether the range of the response options is wide enough (5-point scale) and if the wording in the response options (“strongly disagree” to “strongly agree”) is a sufficient description. It could be interesting to expand the number of response options but, arguably, there could be obstacles with revising such a widely used and widespread instrument.

Regarding the high mean scores for both the total sum and the items of the SUS, it should be noted that the population had a mean age of 33 years and are, therefore, used to digital solutions. This could have influenced their experience of the app. Therefore, the high item scores of the SUS could also indicate a product that is perceived to have usability for this group. A recommendation for future studies would be to test the instrument in other contexts and with different age groups. This app was designed to support users who were in exactly the same situation as the participants in this study: parents in a specific situation. Going home with a child after hospitalization can be stressful; the parents in this study reported this eHealth solution as supportive [46]. The SUS has previously been tested for its sensitivity in different digital solutions and has shown a high scoring (ceiling effect) within the best-of-class products [44].

The Swedish government has envisioned Sweden as a leading country of eHealth in the near future. It has declared the need for individual users to act as the cocreators of such solutions [30]. Within eHealth, there has already been a call for a participatory design to increase the equality of access to digital solutions [47]. Even though digital technology and eHealth are intended to enhance access to health care, for example, despite geographical distances. It may also result in the opposite, as people have different knowledge in using digital solutions. For example, younger people tend to use and access digital solutions differently than older people, which is why solutions should be adapted to the intended end users [48]. In this study, the population was younger, as previously discussed, which could have influenced the result. To have a project design of cocreation often means that end users are involved from the start of the project as collaborators. Coproduction, on the other hand, often involves the end users during the implementation phase [49]. Arguably, the end users are invaluable in all steps of the process, and the usability of products needs to be evaluated where instruments such as the SUS can be useful [50]. This study could therefore be regarded as enhancing the possible participation of end users in the future development of digital solutions and eHealth in a Swedish context.

In conclusion, this study indicates that this developed and psychometrically tested Swedish version of the SUS can be recommended for use within the Swedish adult context.

### Limitations

There are some methodological challenges in this study. First, the sample could be regarded as small (N=62), although it was sufficient according to the recommendations [31,33]. A larger sample might enable different methods of analysis, such as Rasch analysis, which might offer deeper psychometric insights.

Second, the 1-year interval for data collection could be considered long in a rapidly changing world. This length of time could influence how the app was perceived. That said, the digital product was not changed to any great extent during this period, so the perception of usability should not have been influenced to any great extent. Additional data collection was carried out after COVID-19 restrictions were essentially withdrawn in Sweden. There is, however, always the possibility of perceptions fluctuating, as the product could be used in various ways according to the circumstances of the parents.

Third, there was an intention to pursue a data quality test for reproducibility as some of the participants answered the SUS twice. Regrettably, the test-retest sample (n=20) was regarded as too small in this study, and such analyses need to be considered in future studies. In addition, the use of imputation can be discussed. There was a low number of missing data, with a noncomplete frequency of only 9 responses in total. The percentage of missing data per item was also low across all items. This resulted in only 6 imputed scores across the items, and based on mean score for the item, this was regarded as not influencing the result noticeably.

### Conclusions

This study presents a new Swedish version of the SUS. It is the first study to carry out a psychometric evaluation of a Swedish SUS, to establish the Swedish version of the SUS as a reliable and valid instrument for assessing system usability. Overall, the psychometric testing showed high data quality, good scaling assumptions, high internal consistency, and fair convergent validity, all of which support the validity and reliability of the new Swedish version. The results from this study are promising. They raise the possibility that the Swedish version of the SUS could be used to evaluate digital health solutions. To further strengthen the usability of the scale, we suggest additional analysis on data that evaluate more complex eHealth technology and include a wider participant age group, both younger and older.

## Abbreviations

COSMIN: Consensus-Based Standards for the Selection of Health Status Measurement Instruments
eHLQ: eHealth Literacy Questionnaire
SUS: System Usability Scale
WHO: World Health Organization
